# The association of deployment and behavioral health problems with positive drug tests among Army members returning from Iraq or Afghanistan

**DOI:** 10.1186/1940-0640-10-S1-A31

**Published:** 2015-02-20

**Authors:** Mary Jo Larson, Beth A Mohr, Rachel Sayko Adams, Thomas V Williams

**Affiliations:** 1 Institute for Behavioral Health, The Heller School for Social Policy & Management, Brandeis University, Waltham, MA, 02453, USA; 2 Defense Health Agency, Department of Defense, Falls Church, VA, 22042, USA

## Background

Problems associated with alcohol and drug use are a critical area for intervention development within the Department of Defense (DoD). Little is known about the impact of deployment on illicit drug use. This study is the first to study drug use problems with objective drug test data among military members returning from deployment.

## Materials and methods

Using longitudinal data from The Substance Use and Psychological Injury Combat Study, we identify predictors of testing positive for one or more drugs post-deployment among 306,345 enlisted Army active-duty (AD) members returning from Iraq or Afghanistan in FY2008–2011. Subsample analyses examine findings from those members who completed a follow-up questionnaire approximately 6 months after the deployment (n = 262846). Urinalysis tests for metabolites of cocaine, heroin, THC, and/or amphetamines are routinely and randomly tested by the military’s drug test program. These data were examined to estimate the percent of the sample with any positive drug test 6 months and up to 3 years post-deployment (follow-up). Demographic and deployment characteristics and self-report of post-deployment problems were examined as potential predictors of a positive drug test.

## Results

Of AD enlisted members returning from deployment, 2.7 percent had a positive drug test during follow-up (95% confidence interval: 2.68%–2.80%), and the median number of specimens tested during the period was four. Members screening positive for behavioral health problems were more likely to have a positive drug test than those who screened negative: PTSD 4.2 percent versus 2.2 percent; depression 4.8 percent versus 2.1 percent; suicide ideation 6.8 percent versus 2.3 percent; and 6+ alcoholic drinks daily 7.5 percent versus 2.3 percent (unadjusted comparisons; p < 0.0001 for all). Multivariate models controlling for demographic characteristics identified the following predictors associated with an increased odds of a positive drug screen: combat specialist occupation, short deployment (11 months or less), or no prior deployment.

## Conclusions

Preliminary findings suggest that enlisted Army active duty members who are at risk for poor outcomes post-deployment can be identified, and there appears to be an increased risk associated with characteristics of the deployment, in addition to demographic characteristics known to be associated with drug use. Early identification and intervention with enlisted members experiencing post-deployment problems may be useful in enhancing health and reducing drug use after deployment.

